# Human Umbilical Cord Blood Mesenchymal Stem Cells Ameliorate Autism‐Like Behaviors in a Valproic Acid‐Induced Mouse Model via the IGF‐1/Akt Signaling Pathway

**DOI:** 10.1002/brb3.71335

**Published:** 2026-03-31

**Authors:** Jie Tian, Hujing Deng, Zhoujing Hu, Guangzhen He, Juan Zhang, Feiyang Jiang, Jinyun Xu, Yong Wu, Hao Jiang, Ruibo Zhang, Lan Ren, Jiaowei Gu

**Affiliations:** ^1^ Department of Pediatrics Taihe Hospital, Hubei University of Medicine Shiyan Hubei China; ^2^ Institute of Pediatric Research Hubei University of Medicine Shiyan Hubei China; ^3^ Hubei Provincial Clinical Research Center For Umbilical Cord Blood Hematopoietic Stem Cells, Taihe Hospital Hubei University of Medicine Shiyan Hubei China; ^4^ Department of Pediatrics, First Clinical School Hubei University of Medicine Shiyan Hubei China

**Keywords:** autism spectrum disorder, cortical, dendrites, human umbilical cord blood mesenchymal stem cells, valproic acid

## Abstract

**Background:**

Autism spectrum disorder (ASD) is a complex neurodevelopmental disorder that significantly impacts children's physical and mental health, yet effective pharmacological treatments remain limited. The primary objective of this study was to investigate the therapeutic effects of human umbilical cord blood mesenchymal stem cells (hUC‐MSCs) on ASD, evaluate the safety profile of hUC‐MSCs, and elucidate their underlying mechanisms and functional roles.

**Methods:**

In this study, we utilized the offspring of pregnant mice exposed to valproic acid (VPA) as an animal model of ASD. At the beginning of 5 weeks of age, 5 × 10^5^ hUC‐MSCs were administered into the lateral ventricles to evaluate their safety profile and elucidate their potential roles and underlying mechanisms. Specifically, we first monitored the growth and overall health status of the mice following hUC‐MSC treatment and assessed potential toxic effects by performing H&E staining on major organs. Second, behavioral analyses were conducted to examine changes in social interaction, repetitive and stereotyped behaviors, and anxiety‐like behaviors in young mice before and after hUC‐MSC intervention. Finally, the mechanisms underlying the therapeutic effects of hUC‐MSCs in ASD were explored using techniques such as RT‐PCR, Western blot analysis, brain tissue staining, and neuron culture experiments.

**Results:**

Here, we demonstrate that hUC‐MSCs effectively mitigate behavioral abnormalities in a VPA‐induced mouse model of autism without notable adverse effects. Mechanistically, hUC‐MSC treatment promotes cortical neuronal dendritic development and restores the phosphorylation levels of insulin‐like growth factor 1 receptor (IGF‐1R) and protein kinase B (Akt). Furthermore, mRNA expression of synaptic plasticity‐associated genes GAP‐43 and SYP, as well as the anti‐inflammatory cytokine IL‐10, was significantly upregulated, while the expression of proapoptotic genes Bax and Caspase‐3, along with pro‐inflammatory cytokines IL‐6 and IL‐1β, was markedly suppressed.

**Conclusions:**

These findings suggest that hUC‐MSCs may exert neuroprotective effects by modulating the IGF‐1/Akt signaling pathway, promoting neuronal development, reducing neuroinflammation, and inhibiting apoptosis, ultimately alleviating core ASD‐like symptoms. The therapeutic benefits may stem from paracrine factors secreted by hUC‐MSCs or their ability to regulate gene expression linked to neuronal development. Our study provides new insights into ASD pathogenesis and highlights the potential of hUC‐MSCs as a novel stem cell‐based therapy for ASD.

## Graphical abstract

Human umbilical cord blood mesenchymal stem cells (hUC‐MSCs) were intraventricularly transplanted into valproic acid (VPA)‐induced autistic mice. hUC‐MSCs significantly ameliorated core ASD‐like behaviors, including social deficits and repetitive behaviors. Mechanistically, hUC‐MSCs promoted cortical neuronal dendritic development and restored IGF‐1R/Akt signaling pathway activation. Additionally, hUC‐MSCs upregulated synaptic plasticity genes (GAP‐43, SYP) and anti‐inflammatory cytokine IL‐10, while downregulating proapoptotic (Bax, Caspase‐3) and pro‐inflammatory factors (IL‐6, IL‐1β). These findings highlight hUC‐MSCs as a promising therapeutic strategy for autism spectrum disorder (ASD) via multifaceted neuroprotective mechanisms.

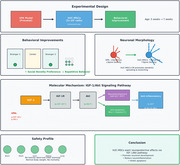

## 1 Introduction

Autism spectrum disorder (ASD) is a neurodevelopmental disorder that greatly affects the physical and mental health of children. According to estimates by the WHO, as of 2024, the global prevalence of autism among children is approximately one in 100. ASD has become one of the most important mental illnesses in children (Hyman et al. 2020). The lack of effective treatments has turned it into a major global public health issue. However, early diagnosis and timely intervention are crucial for enhancing the rehabilitation outcomes of ASD individuals (Li et al. 2021). Currently, the treatment of ASD typically involves a multidisciplinary comprehensive intervention strategy. In clinical practice, this primarily encompasses a combination of pharmacotherapy, behavior modification, educational training, and physical therapy (Genovese and Butler 2020; Hirota and King 2023). To date, no globally approved medications specifically target the core symptoms of ASD. Clinically, antipsychotics, antidepressants, oxytocin, or vasopressin receptor antagonists, and nutritional supplements are predominantly utilized to manage complications and associated symptoms (Turner 2020). It is important to emphasize that these drugs remain under investigation, with their efficacy and safety yet to be fully established. Furthermore, behavioral interventions such as applied behavior analysis and cognitive‐behavioral therapy represent some of the safest and most effective approaches for ASD‐related behavioral interventions and rehabilitation, yet, some concerns have been raised by several scholars regarding the potential for these methods to overly control autistic behaviors without adequately respecting the unique needs of autistic individuals (Bottema‐Beutel and Crowley 2021). Additionally, while educational training and physical therapy may contribute to overall development, they demonstrate limited impact on alleviating the core symptoms of ASD in children. In summary, these treatments have limited benefits and cannot fundamentally treat children with ASD (Wang et al. 2023; Pistollato et al. 2020). Consequently, identifying a therapeutic approach with high efficacy, minimal side effects, and robust safety remains an unresolved challenge in ASD research.

In recent years, the application of mesenchymal stem cells in the treatment of nervous system disorders has expanded significantly, offering a promising new avenue for these challenging conditions. Current research indicate that human umbilical cord blood mesenchymal stem cells (hUC‐MSCs) possess significant potential for the treatment of ASD. hUC‐MSCs are highly favored due to their ease of acquisition, robust expansion capacity, low immunogenicity, and minimal ethical concerns (Chen et al. 2010). hUC‐MSCs have immunomodulatory effects and can inhibit inflammatory response by secreting anti‐inflammatory factors and regulating immune cell function (Joel et al. 2019). Transplanted hUC‐MSCs release immunosuppressive substances like IL‐10 and TGF‐β, enabling nonspecific immune regulation by inhibiting dendritic cell maturation and antigen presentation. Peripheral blood mononuclear cells from ASD patients excessively produce IL‐1β, causing prolonged immune changes. MSCs' immunosuppressive effects, which inhibit TNF‐α and IFN‐γ production while enhancing IL‐10 levels, can rectify this imbalance (Musiał‐Wysocka et al. 2019; Qu et al. 2022). Due to its unique biological characteristics and therapeutic potential, hUC‐MSCs will provide a more effective treatment strategy for the treatment of neurodevelopmental disorders ASD. In this study, we employed offspring of pregnant mice exposed to valproic acid (VPA) as a model of ASD by injecting hUC‐MSCs into the lateral ventricle, to investigate their therapeutic effects on ASD, evaluate the safety profile of hUC‐MSCs, and elucidate their potential roles and underlying mechanisms. This will provide theoretical support for the research of hUC‐MSCs therapy in clinical ASD treatment and facilitate the further application of hUC‐MSCs in treating ASD.

## 2 Materials and Methods

### 2.1 Animals

Adult Kunming mice, both male and female, weighing 40 ± 5 g, were sourced from the Laboratory Animal Center, Hubei University of Medicine, Shiyan, China. Mice were paired overnight, and gestation Day 1 (GD 1) was identified by detecting sperms in vaginal smears. Mice were housed in a regulated setting with a 12‐h light/dark cycle, temperatures ranging from 19°C–22°C, and unrestricted access to food and water.

### 2.2 Modeling and Treatment With Valproate Sodium Salt (VPA)

All procedures adhered to the university's ethical committee guidelines. The pregnant mice were divided into two groups: one group was administered physiological saline (10 mL*/*kg) on gestation Days 12 and 13, while the other group received an intraperitoneal injection of sodium valproate (300 mg/kg) on those same days. The born pups from the VPA‐exposed group were assigned to either a sham group or a treatment group. The behavior tests were conducted on pups in the VPA‐exposed group at postnatal Day 29, and those exhibiting autism‐like behaviors were selected for subsequent experiments. Thus, the study comprised three groups: (1) control group, treated prenatally with physiological saline; (2) VPA group, serving as the sham group; and (3) MSCs group, treated with hUC‐MSCs. At the beginning of 5 weeks of age, in the VPA + hUC‐MSCs group, mice received intraventricular administration of hUC‐MSCs (5 × 10^5^), while the control and the sham group underwent craniotomy without any substance being administered. After a 2‐week recovery period, behavioral examinations were conducted when the mice reached 7 weeks of age. Behavioral tests were followed by histological evaluations.

### 2.3 Behavioral Tests

#### 2.3.1 Three‐Chamber Social Test

All behavioral tests were recorded using a video camera from SansBio, China. Social dysfunction can be evaluated using a three‐chamber social test at postnatal Day 29. The experimental apparatus consisted of a rectangular acrylic glass structure measuring 60 × 45 × 25 cm featuring two partition walls that evenly divided it into three sections. The mice's access to different rooms was controlled by sliding doors in each partition wall. In the left and right chambers, two cylindrical cages were placed, each with a diameter of 20 cm and a height of 30 cm. The test comprised two phases; initially, it involved a social stimulus and a non‐social stimulus (NS), represented by a cartoon plastic toy resembling the pups in size. In the second phase, one NS was substituted with a social stimulus, specifically an unfamiliar mouse matched for age and gender. Pups acclimated to the experimental environment 1 day prior. Mice were given 10 min to freely acclimate to the environment with two empty cages positioned in the left and right chambers. Social competence was assessed by placing various stimuli in cages within the left and right chambers. The animals were placed in the center of the middle chamber and given 10 min per stage to explore, with a 5‐min break between tests when the pups were returned to the center of the middle chamber. The timing of the mice's sniffing behavior was marked as the recording index. After each test, the area was cleaned with 75% ethanol. A blinded researcher conducted the results analysis.

#### 2.3.2 Open Field (OF)Test

The tests were performed on the 29th and 49th days after birth (2 weeks after stem cells therapy). The experimental apparatus consisted of a rectangular acrylic glass structure measuring 45 × 45 × 25 cm. Mice were positioned at the square's center and observed for 6 min. The time and distance mice spent in the central area were recorded. After each test, the OF was cleaned with 75% ethanol to eliminate any odor interference.

#### 2.3.3 Marble‐Burying Test

Each mouse was housed in a separate cage measuring 30 cm × 30 cm × 20 cm, with a 5 cm layer of corncob bedding. The cage holds nine glass marbles, each with a diameter of 2.5 cm, organized in a 3 × 3 × 3 configuration. The mice were alone in cages, and the number of glass marbles buried within 10 min was documented. A marble was deemed buried if corncob bedding covered at least two‐thirds of its volume.

### 2.4 Western Blot

After the behavioral tests, the mice were euthanized following anesthesia administration. The cortex was quickly removed and homogenized in RIPA lysis buffer mixed with the protease and phosphatase inhibitor cocktail (Beyotime, China) following the infusion of 0.9% saline. The homogenate was then centrifuged at 12,000 rpm at 4°C for 10 min to collect the supernatant and store it at −80°C. We used the BCA protein assay kit from Beyotime, China to determine the protein concentration. Next, the protein were loaded onto denatured 10% SDS‐PAGE gels and subsequently transferred to PVDF membranes (Millipore). The membranes were left at normal temperature for 1 h with 5% bovine serum albumin. The membranes were incubated for 12–15 h with primary antibodies: anti‐IGF‐1R (1:1000, Abcam, ab182408), anti‐phosphorylated‐IGF‐1R (1:1000, Cell Signaling Technology, 3021s), anti‐Akt (1:1000, Cell Signaling Technology, 9272), anti‐phosphorylated‐Akt (1:1000, Cell Signaling Technology, 4060), and anti‐GAPDH (1:10000, Abcam, ab8245) at 4°C. The membranes were exposed to enzyme‐labeled secondary antibodies (1:2000, Beyotime, China) for 2 h at normal temperature. Afterwards, we used Immobilon Western Chemiluminescent HRP Substrate (Millipore) detected protein bands. Image J was utilized for the quantitative analysis of the strips.

### 2.5 Quantitative Real‐Time Polymerase Chain Reaction

The expression levels of SYP, GAP‐43, IL‐1β, IL‐6, IL‐10, Caspase‐3, and Bax genes were evaluated using qRT‐PCR. The mice brains from each group were quickly extracted and immersed in chilled PBS, and the whole cortex was dissected, quickly frozen in liquid nitrogen, and kept at −80°C until needed. The total RNA of mice cerebral cortex was extracted by Trizol method. RNA quantification was conducted with a NanoDrop 1000 instrument (Thermo Fisher Scientific). The HiFiScript cDNA Synthesis Kit (CWBIO, China) was used to synthesize cDNA. The qRT‐PCR was performed using UltraSYBR mixture (CWBIO, China) on a Bio‐Rad CFX96 PCR system (Table 1). Afterwards, gene expression levels were evaluated using the ΔCT method, normalized against the geometric mean of GAPDH housekeeping genes, and compared to the control intact group. The expression levels of target genes were shown as fold changes.

**TABLE 1:** Primers utilized for qRT‐PCR.

Primer name	Sequence	Primer length	Tm melt (°C)
GAP‐43	Forward CTCCAACGGAGACTGCAGAA Reverse CCTGTCGGGCACTTTCCTTA	87	59.68
SYP	Forward CTCCTCAGCCCCTATCGGTT Reverse AGTCACATCGACCCACTCCA	110	60.76
Bax	Forward GAGAGGTCTTCTTCCGGGTG Reverse CTGATCAGCTCGGGCACTTT	136	60.39
Caspase‐3	Forward TGGCTTGCCAGAAGATACCG Reverse GACTGGATGAACCACGACCC	109	60.39
IL‐6 (Noshadian et al. 2024)	Forward CCACTGCCTTCCCTACTTCAC Reverse CAGTGCATCATCGCTGTTC	185	60.34
IL‐1β (Noshadian et al. 2024)	Forward TGGCTGTGGAGAAGCTGTGG Reverse GCAGTGCAGCTGTCTAATGG	176	62.40
IL‐10 (Noshadian et al. 2024)	Forward CAATAACTGCACCCACTTCCC Reverse CTTGGCAACCCAAGTAACCC	164	59.18

### 2.6 Cell Culture

Pregnant female mice from the VPA‐exposed group at 18.5 days of embryonic development were anesthetized with CO_2_. Following uterine exposure, embryos were collected and thoroughly rinsed with chilled HBSS. Brains were dissected, and connective tissue was meticulously removed under a dissecting microscope, then, the cerebral cortex was harvested and broken down in HBSS containing 2 mg/mL papain and 10 mg/mL deoxyribonuclease, both from Absin, China, for 20 min at 37°C. The cell suspension in the supernatant was transferred to a 15 mL centrifuge tube and spun at 2500 rpm for 3 min. Following the removal of the supernatant, 4 mL of medium was used to gently resuspend the cells. The cell suspensions were transferred to a 50 mL centrifuge tube combined with Gibco Neurobasal Plus medium, supplemented with Gibco B‐27 Plus, Gibco GlutaMAX, and penicillin/streptomycin, and then seeded onto poly‐*D*‐lysine‐treated 12‐well or 24‐well plates. The hUC‐MSCs were supplied by Beike Biotechnology. All cells were cultured under 37°C, 90% humidity, and 5% CO_2_.

### 2.7 Immunofluorescence

On the 4th day of culturing neurons, the slides in the culture plate underwent three washes with 1× PBS, each for 5 min. Afterward, the cells were fixed using 4% paraformaldehyde at normal temperature for 40 min. After fixation, the slides underwent three 5‐min washes with 1× PBS, followed by a 1‐h incubation at normal temperature in a solution of 5% BSA and 0.25% Triton X‐100. The slides were subsequently incubated overnight at 4°C with the primary antibodies, including a murine monoclonal antibody against MAP‐2 protein (1:500) and a rabbit anti‐GFAP antibody (1:500). The following day, the slides were rinsed three times with 1× PBS. Secondary antibodies, Alexa Fluor 488 Goat Anti‐Mouse IgG (H + L) and Alexa Fluor 568 Goat Anti‐Rabbit IgG (H + L), were diluted at a 1:2000 ratio in 5% BSA and incubated in the dark at normal temperature for 2 h. The nuclei were stained with DAPI (Beyotime, China) for 10 min, followed by three 5‐min washes with 1× PBS. The slides were then examined using a fluorescence microscope.

### 2.8 Golgi Staining

The FD Rapid GolgiStainTM Kit (FD NeuroTechnologies) was used to perform Golgi impregnation on whole brains from three groups of mice, adhering to the manufacturer's instructions. Mice were euthanized in a 4% CO_2_ chamber for 5 min at the specified time, after which their brains were promptly extracted and rinsed with tap water. We soaked each group of brains in a Golgi–Cox solution containing potassium dichromate, mercury chloride, and potassium chromate. After 16 h of initial immersion, the solution mixture was replaced and subsequently stored in the dark at normal temperature for 2 weeks. Following immersion in solution, the embedded brains were placed in a cryoprotectant solution and kept at 4°C in the dark for a minimum of 1 week prior to sectioning. Coronal plane brain slices were sectioned at a thickness of 100–200 µm using a microtome. The brain slices were set on slides coated with gelatin and allowed to air dry in the dark at normal temperature overnight before undergoing further processing. Once dried, the sections were washed with tap water, stained in a developing solution, and then dehydrated through a series of ethanol concentrations: 50%, 75%, 95%, and 100%. They were then defatted in xylene and mounted on coverslips with neutral gum (Beyotime, China). Images were captured using the oil immersion lens of an Olympus microscope. ImageJ software was used to measure the number and size of spines, as well as the dendrite length, on cortical pyramidal neurons.

### 2.9 Statistical Analysis

The data were processed using GraphPad Prism 8 from San Diego, California, USA. For comparisons between groups, a standard unpaired *t*‐test was used. Experiments with three and more groups, one way ANOVA followed by post hoc Tukey multiple comparisons were performed. The data are presented as mean ± SEM, with a *p*‐value of less than 0.05 indicating statistical significance.

## 3 Results

### 3.1 Treatment With hUC‐MSCs Alleviates VPA‐Induced Autism‐Like Behaviors

**FIGURE 1:**
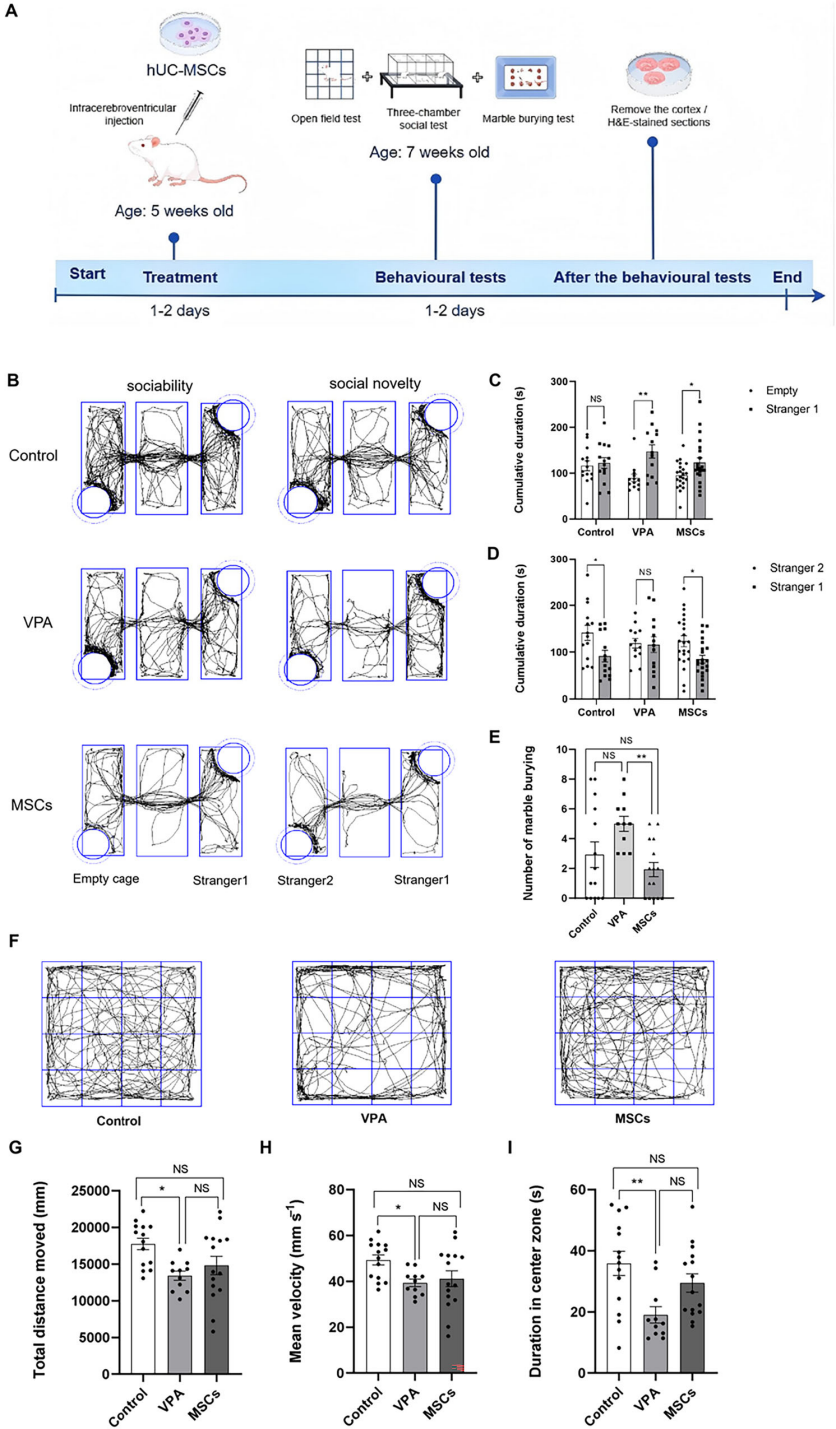
The hUC‐MSCs treatment significantly attenuates autism‐spectrum behaviors in VPA‐induced mice. (A) Flow chart for the study procedure. (B) Three‐chamber test; *n* = 14 (control), 13 (VPA), 22 (MSCs). (C) Duration of sociability. (D) Duration of social novelty. (E) Marbles bury test; *n* = 14 (control), 11 (VPA), 15 (MSCs). (F) The open field test; *n* = 14 (control), 11 (VPA), 15 (MSCs). (G–I) The total distance, average speed, and center duration were analyzed for comparison. Results are shown as mean ± SEM. *p < 0.05; **p < 0.01; NS means not significant.

At first, we proceeded with a three‐chamber social test to evaluate the sociability and the social novelty of mice. In sociability tests, both the VPA (sham group) and MSCs (VPA + hUC‐MSCs group) demonstrated a preference for spending more time with social targets (Stranger 1) over an inanimate object (Figure 1B,C). In social novelty tests, the control and MSCs exhibited a preference for new social targets (Stranger 2) over familiar ones (Stranger 1) (Figure 1B,D). No difference was observed in the time spent between Stranger 2 and Stranger 1 in VPA (Figure 1B,D). These data suggest that the social novelty was significantly enhanced in ASD mice treated with hUC‐MSCs. We conducted an assessment to examine potential repetitive and stereotyped behaviors. In marble burying tests, VPA bury more marbles compared to control, while the MSCs buried significantly fewer beads (Figure 1E). We employed the OF test to assess if MSCs alleviate anxiety‐like behaviors in mice. In OF tests, MSCs exhibited comparable total distance moved and mean velocity to VPA (Figure 1F–H). Although MSCs tended to spend more time in the central region than VPA, this difference was not statistically significant (Figure 1F,I). The findings suggest that hUC‐MSCs minimally impact anxiety‐like behavior in mice.

### 3.2 hUC‐MSCs Promote the Development of Cortical Neuronal Dendrites

**FIGURE 2:**
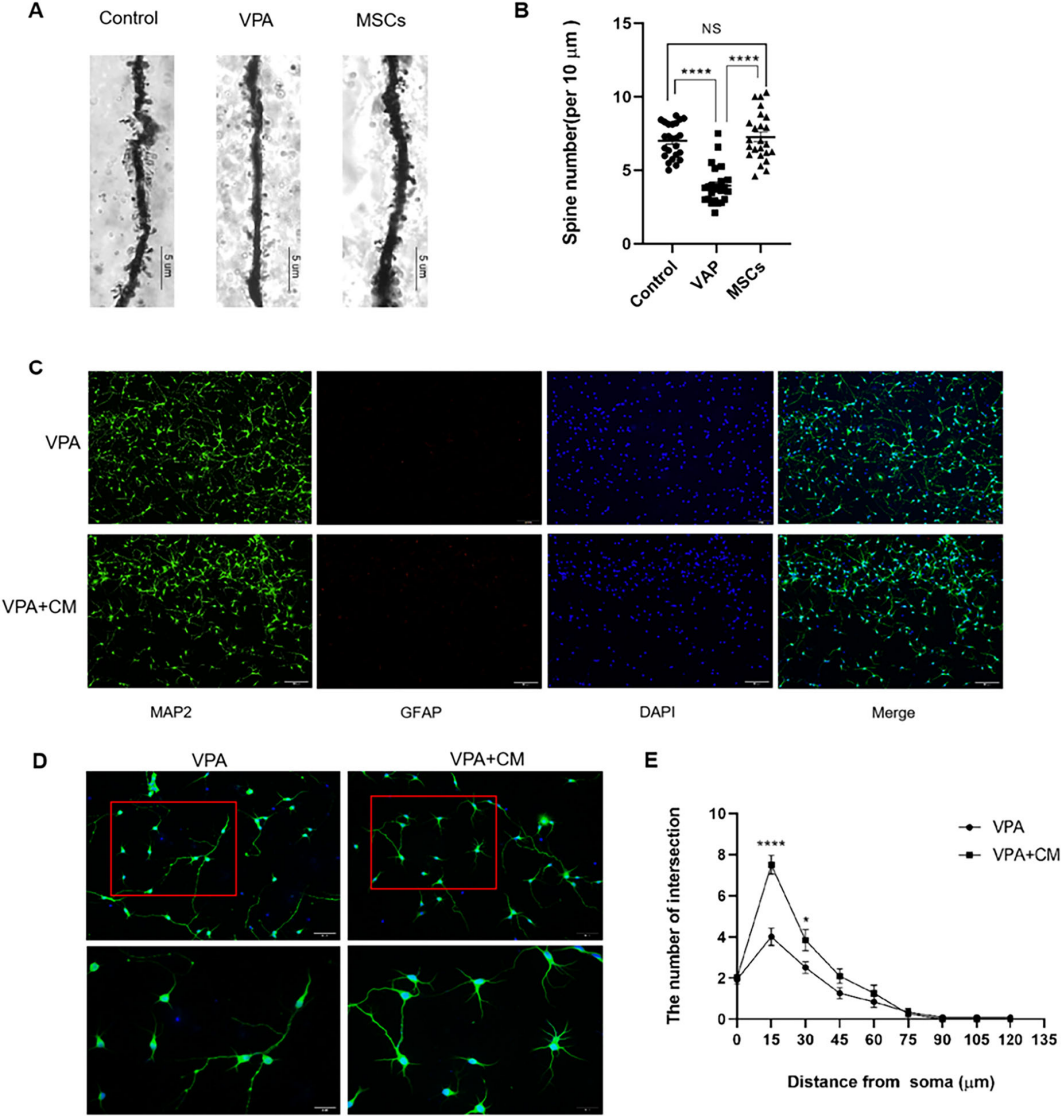
hUC‐MSCs promote the development of cortical neuronal dendrites. (A) Golgi staining of the cortical neurons. The scale bar represents 5 µm at 100× magnification. (B) The dendritic spine within 24 dendritic segments from three mice in each group were quantitatively analyzed. (C) Representative images of primary cultured neurons are shown with MAP2 in green, GFAP in red, and DAPI in blue. The scale bar represents 100 µm at 10× magnification. (D) Immunofluorescence staining of MAP2 in primary neurons co‐cultured with hUC‐MSCs‐CM is shown; scale bars represent 50 µm at 20× magnification and 20 µm at 40× magnification. (E) Sholl analysis was used to evaluate the dendritic complexity of cortical neurons, with 12 neurons analyzed from three mice per group. Data are presented as mean ± SEM. *p < 0.05; **p < 0.01; NS indicates not significant.

Given the common reports of dendritic dysplasia in mouse models related to ASD (Yan et al. 2022; Tong et al. 2019), we applied Golgi staining to brain tissues. Neurons from the cortex of VPA showed a reduced spine density compared to the control, which could be saved by hUC‐MSCs therapy (Figure 2A,B). To investigate the relative contributions of paracrine signaling mechanisms, we adopted a complementary experimental approach—specifically, treatment with conditioned medium (CM)—to assess the effects mediated by soluble factors. We investigated the effect of hUC‐MSCs on neuronal growth by culturing primary neurons from VPA‐exposed embryos with hUC‐MSCs‐CM. We subsequently dyed the neuronal cells with anti‐MAP2, a common dendrite marker for neurons (Figure 2C). The results indicated that hUC‐MSCs‐CM promoted the germination and branching of neuronal dendrites, and the neuronal complexity was higher than that in the group without hUC‐MSCs‐CM (Figure 2D,F). We propose that hUC‐MSCs‐CM may enhance the growth of cortical neuronal dendrites through its neurotrophic effects.

### 3.3 Potential Mechanism of hUC‐MSCs in ASD Treatment

**FIGURE 3:**
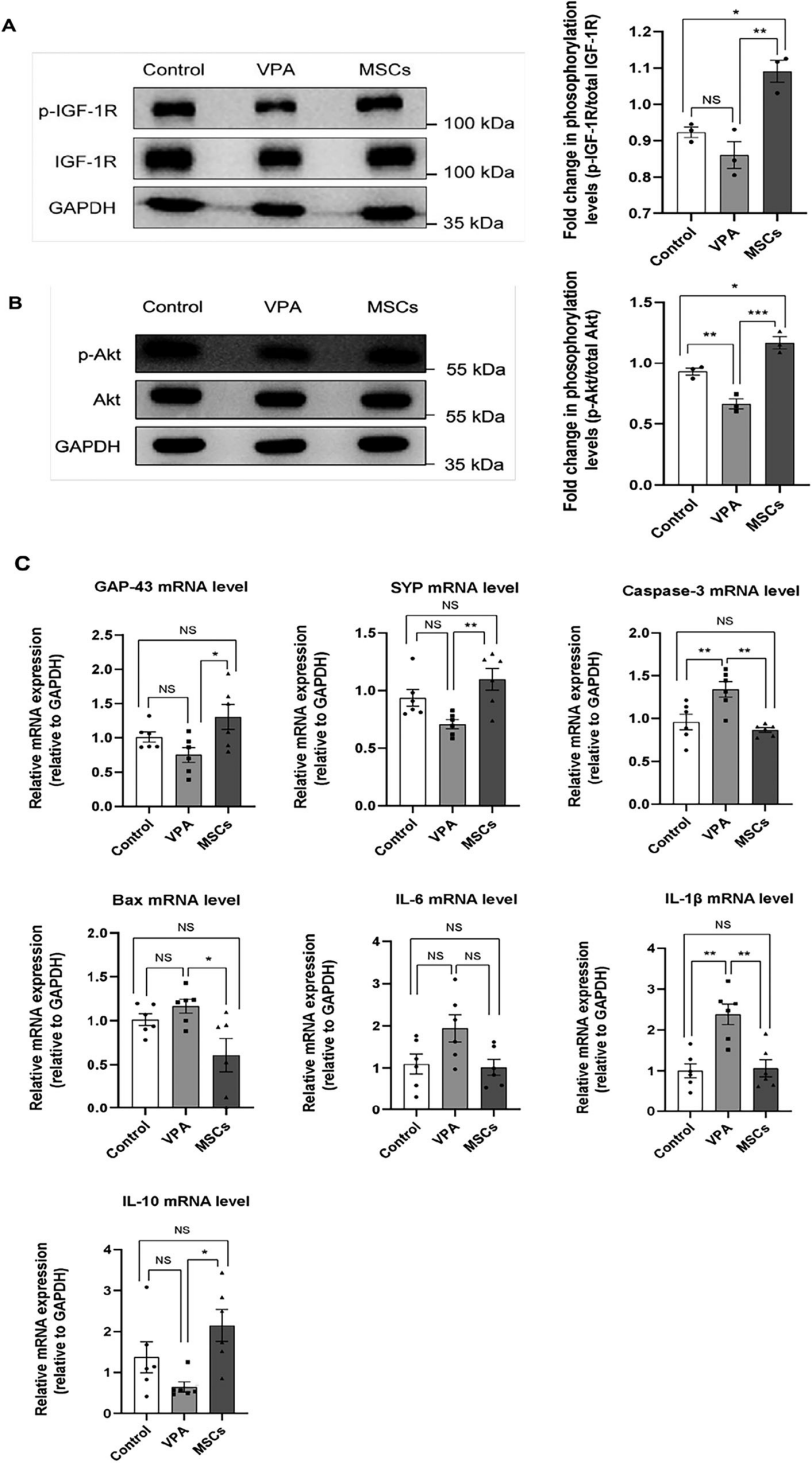
In the cerebral cortex of VPA‐induced ASD mice, hUC‐MSCs transplantation normalizes p‐IGF‐1R/IGF‐1R and p‐Akt/Akt ratios, enhances neuronal development gene expression, and suppresses inflammation and apoptosis‐related gene expression. (A) Representative immunoblots and quantitative analysis of phosphorylated IGF‐1R (p‐IGF‐1R) and total IGF‐1R protein levels in cortical tissue lysates. The ratio of p‐IGF‐1R to total IGF‐1R was determined by densitometry (*n* = 3 per group). (B) Representative immunoblots and quantitative analysis of phosphorylated Akt (p‐Akt) and total Akt protein levels in cortical tissue lysates (*n* = 3 per group). (C) Quantification of mRNA levels for GAP‐43, SYP, Caspase‐3, Bax, IL‐6, IL‐1β, and IL‐10 in the cortex (*n* = 6 per group). Data are presented as mean ± SEM, with each dot on the graph representing an individual biological replicate. *p < 0.05; **p < 0.01; NS indicates not significant.

To elucidate the functional recovery observed in the VPA + hUC‐MSCs group, we explored the molecular processes that contribute to the therapeutic benefits of hUC‐MSCs. Yang et al. (2024) recently demonstrated that IGF‐1R significantly contributes to ASD pathogenesis, with its rare variants and disrupted regulatory networks potentially impacting neurodevelopment and neuronal function. Our study observed reduced levels of p‐IGF‐1R and p‐Akt in mice with VPA‐induced autism. The treatment with hUC‐MSCs restored the altered expressions of p‐IGF‐1R and p‐Akt (Figure 3A,B). Prior research suggest that the effectiveness of hUC‐MSCs transplantation may be due to their immunomodulatory, anti‐inflammatory, and neurotrophic properties (Li et al. 2023). Our findings indicate that hUC‐MSCs treatment restored the reduced mRNA expression levels of GAP‐43, SYP, and IL‐10 in the cortices of VPA mice (Figure 3C). In VPA mice treated with hUC‐MSCs, the mRNA expression levels of Bax, Caspase‐3, IL‐6, and IL‐1β in the cortex were reduced (Figure 3C). This study suggest that the transplantation of hUC‐MSCs may be effective due to their anti‐inflammatory and neurotrophic properties. These findings indicate that the IGF1/Akt pathway could serve as a molecular target for both the pathogenesis and treatment strategies of ASD.

### 3.4 hUC‐MSCs Treatment Exhibits No Detectable Adverse Effects

**FIGURE 4:**
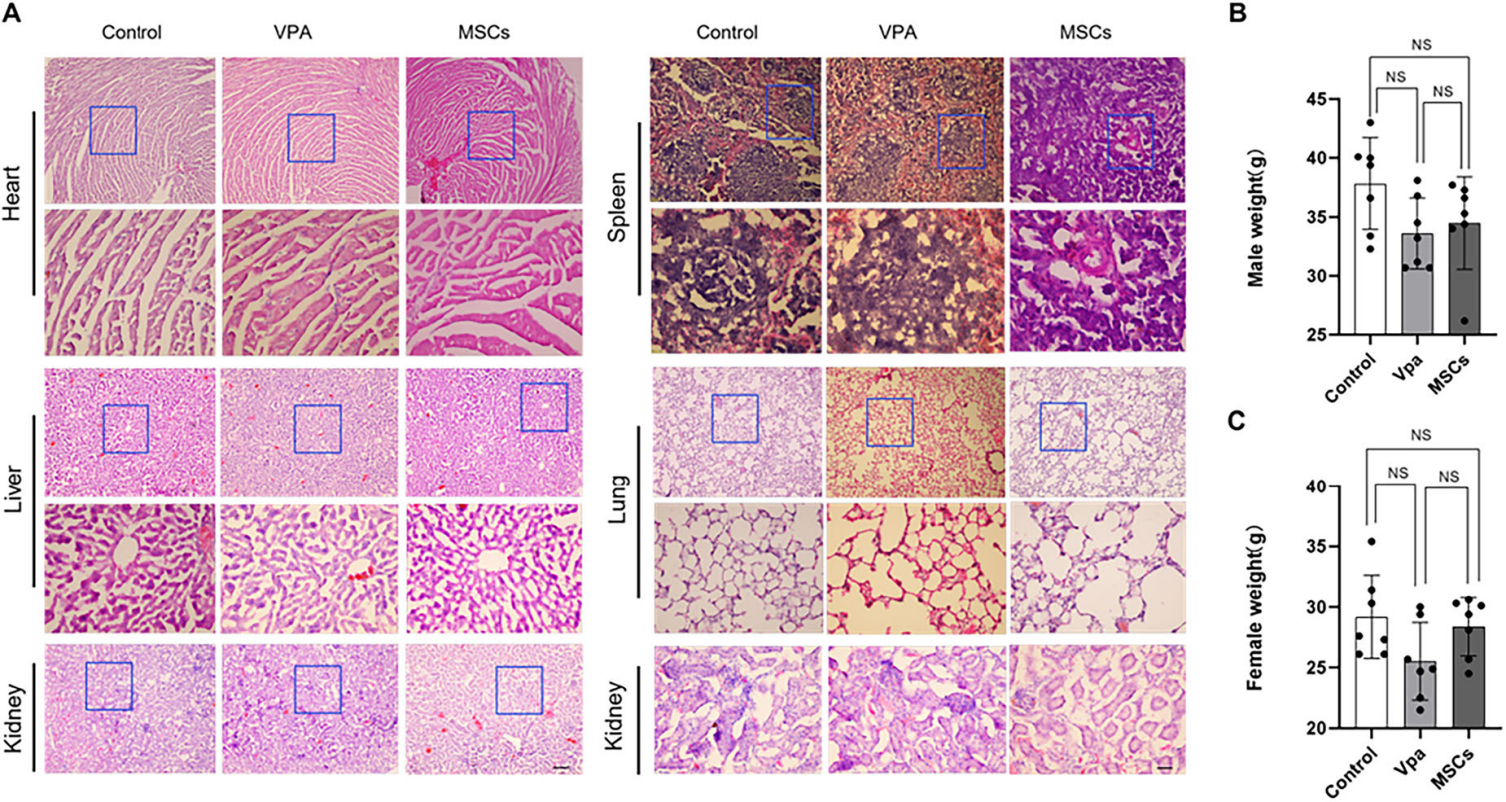
The hUC‐MSCs shows no clear in vivo toxicity. (A) Representative micrographs of H&E‐stained sections 2 weeks after surgery; scale bars represent 100 µm at 10× magnification and 20 µm at 40× magnification. (B,C) Average body weight of each group 2 weeks after surgery. *n* = 7 (control), 7 (VPA), 7 (MSCs). Data are presented as mean ± SEM. *p < 0.05; **p < 0.01; NS indicates not significant.

The potential toxicity of hUC‐MSCs was subsequently evaluated in a comprehensive manner. After 2 weeks of treatment with hUC‐MSCs, no adverse effects were observed on the growth or overall health of the mice, as evidenced by consistent body weight (Figure 4B,C) and normal external appearance. No mortality was observed among the recipient mice following exposure to the administered hUC‐MSCs doses. Furthermore, macroscopic examination revealed no histomorphological alterations attributable to hUC‐MSCs treatment. Toxicity to major organs was also assessed using H&E staining, which showed no significant abnormalities or discernible organ damage across all analyzed organs, irrespective of the experimental group (Figure 4A). These findings provide compelling evidence that hUC‐MSCs exhibit minimal tissue toxicity. While no immediate safety concerns were identified, we acknowledge that detailed safety validation—including cell residual statistics, long‐term survival tracking, and comprehensive biodistribution analysis—was not performed. Given the established preclinical safety profile of human umbilical cord blood‐derived MSCs in existing literature, and considering the exploratory nature of this study, we believe our preliminary safety observations are acceptable. Future translational studies should incorporate dedicated safety endpoints, including quantitative cell tracking and long‐term follow‐up.

## 4 Discussion

ASD is a neurodevelopmental disorder, and this neurodevelopmental abnormality typically emerges in early childhood and may persist throughout life, with a long‐standing lack of effective therapeutic drugs. In this study, we employed offspring of pregnant mice exposed to VPA as a model of ASD. By injecting hUC‐MSCs into the lateral ventricle, to investigate their therapeutic effects on ASD, we demonstrate that hUC‐MSCs effectively mitigate behavioral abnormalities in a VPA‐induced mouse model of autism without notable adverse effects.

ASD is closely related to abnormalities in brain neural network connectivity, and dendritic developmental defects may be one of the key factors leading to such abnormalities (Genovese and Butler 2023). Dendrites, essential for neuronal information reception, may contribute to ASD pathogenesis by influencing synaptic transmission and neural circuit formation through changes in their morphology and function. Postmortem analyses of ASD patients reveal a notable rise in dendritic branching and complexity in brain areas like the prefrontal cortex, temporal lobe, and cerebellum. However, some studies have also reported decreased dendritic branching, indicating that the heterogeneity of ASD may be reflected in different brain regions or subtypes (Jin et al. 2019; Sato and Uono 2019). Dendritic spines, a key component of postsynaptic structures, may lead to defects in synaptic pruning in later development due to excessive generation in early development, ultimately causing an imbalance in neural networks (Chafee and Averbeck 2022). In this study, by conducting Golgi staining on the cerebral cortex of mice, we demonstrated that hUC‐MSCs facilitate the development of dendrites in cortical neurons of ASD mice. To our knowledge, this is the first time that the impact of hUC‐MSCs on dendritic development in cortical neurons of ASD has been visualized and confirmed at the microscopic level. In our study, hUC‐MSCs‐CM greatly enhances the sprouting and branching of neuronal dendrites, indicating that hUC‐MSCs may secrete certain factors that promote neuronal development or induce changes in the expression of related genes.

In addition, our study demonstrates that hUC‐MSCs treatment restored p‐IGF‐1R and p‐Akt expression and significantly increased mRNA levels of GAP‐43, SYP, and IL‐10, also, in VPA mice treated with hUC‐MSCs, mRNA expression of Bax, Caspase‐3, IL‐6, and IL‐1β was inhibited. These findings suggest that the IGF‐1/Akt pathway could be a molecular target for ASD pathogenesis and treatment strategies. IGF‐1 is a key regulator of cell proliferation and differentiation, essential for individual growth and development. IGF‐1 activates the IGF‐1R receptor, triggering the PI3K/Akt signaling pathway, which inhibits proapoptotic proteins like Bax and Bad while promoting the expression of antiapoptotic proteins such as Bcl‐2 (Xu et al. 2025). The phosphorylation of Akt can directly inactivate the apoptosis‐related enzyme caspase‐9, enhancing neuronal resistance to oxidative stress and metabolic damage. Studies have shown that in diabetic encephalopathy models, decreased activity of the IGF‐1/Akt pathway is closely related to increased neuronal apoptosis (Kaur and Aran 2025). IGF‐1 activates the mTOR pathway through Akt, promoting the extension of neuronal axons and dendrites and regulating the directed differentiation of neural stem cells into functional neurons (Banjac et al. 2024), while blocking Akt activity leads to abnormal neuronal morphological development. The IGF‐1/Akt pathway integrates growth signals with metabolic demands to achieve multidimensional regulation in neurodevelopment, and its functional imbalance may lead to developmental abnormalities or neurodegenerative diseases. Targeting this pathway could be valuable for treating neurological diseases. Currently, clinical trials have indicated that IGF‐1 exhibits potential therapeutic effects on neurodevelopmental disorders and can ameliorate pathophysiologic behavioral abnormalities (Vahdatpour et al. 2016). In a recent trial involving children with autism, IGF‐1 significantly alleviated stereotyped behavior and hyperactivity (Kolevzon et al. 2022). This study provides preliminary evidence that hUC‐MSCs may exert therapeutic effects in ASD via the IGF‐1/Akt pathway. However, we acknowledge several limitations. First, direct multi‐omics evidence (e.g., RNA‐Seq or proteomics) is lacking; future studies incorporating transcriptomic or proteomic profiling will be essential to comprehensively elucidate the global molecular mechanisms underlying MSC action. Second, protein‐level validation of key inflammatory mediators (e.g., BAX, Caspase‐3, IL‐6, IL‐1β) was not performed due to limited sample availability, rendering our conclusions regarding anti‐inflammatory mechanisms preliminary. Third, the VPA‐induced model, while valuable for studying neurodevelopmental alterations, lacks comprehensive parameters bridging behavior, structure, function, and cognition, and does not capture the genetic heterogeneity characteristic of clinical ASD. Consequently, this model cannot fully replicate the multifactorial nature of autistic patients, in whom genetic and environmental factors play complex interacting roles. Moving forward, we aim to translate these findings into preliminary clinical investigations of hUC‐MSCs for treating ASD in pediatric populations.

## 5 Conclusion

Our study suggest that hUC‐MSCs can significantly relieve core social dysfunction in the VPA‐induced autism model by promoting neurodevelopmental, anti‐inflammatory, and antiapoptotic effects via the IGF‐1/Akt signaling pathway. These findings provide valuable insights for further understanding the pathogenesis of ASD and the clinical stem cells treatment of ASD.

## Author Contributions

J.G. and J.T. conceived and designed the study. J.T., H.D., F.J., G.H., J.X., and Y.W. conducted the experiments and participated in data interpretation. Z.H, J.Z., H.J., R.Z., and L.R. assisted in reviewing and proofreading the manuscript. J.T. drafted the manuscript with input from all co‐authors. All authors made distinct contributions to the presented work, and reviewed and approved the final version of the manuscript.

## Funding

This research received funding jointly supported by the Hubei Provincial Natural Science Foundation and Shiyan Innovation and Development Joint Foundation of China (2025AFD215) and the Open Fund Hubei Provincial Clinical Research Center for Umbilical Cord Blood Hematopoietic Stem Cells, Taihe Hospital (2024SCOF010 and 2025SCOF017).

## Ethics Statement

The ethics approval for the research project titled “Research on the Therapeutic Effects and Underlying Mechanisms of Human Umbilical Cord‐Derived Mesenchymal Stem Cells in Autism Mouse Models” was granted by the Medical and Laboratory Animal Ethics Committee of Hubei University of Medicine, Shiyan, Hubei, China. The approval number for this project is Hubei University of Medicine F‐2025‐Real‐040, and the date of approval is April 30, 2025. All animals involved in the study were handled in accordance with ethical guidelines, and their care and use were approved by the Medical and Laboratory Animal Ethics Committee of Hubei University of Medicine.

## Conflicts of Interest

The authors declare no conflicts of interest.

## Data Availability

The data that support the findings of this study are available from the corresponding author upon reasonable request.
